# Draft Genome Sequence of an Extensively Drug-Resistant Mycobacterium tuberculosis Clinical Isolate, 3485_MTB, from Nur-Sultan, Kazakhstan

**DOI:** 10.1128/MRA.00025-20

**Published:** 2020-03-05

**Authors:** Pavel Tarlykov, Sabina Atavliyeva, Arike Alenova, Yerlan Ramankulov

**Affiliations:** a National Center for Biotechnology, Nur-Sultan, Kazakhstan; b National Scientific Center for Phthisiopulmonology, Almaty, Kazakhstan; Loyola University Chicago

## Abstract

Here, we report the draft genome sequence of an extensively drug-resistant Mycobacterium tuberculosis clinical isolate, 3485_MTB, from Nur-Sultan, Kazakhstan. The genome sequence is composed of 4,836,003 bp. The genome will provide more data on the genetic variations occurring in local drug-resistant isolates.

## ANNOUNCEMENT

In 2019, the World Health Organization included Kazakhstan on a list of 30 countries with large burdens of drug-resistant tuberculosis (1). A total of 4,800 cases of multidrug-resistant (MDR) tuberculosis were registered in Kazakhstan in 2018. The problem of MDR or extensively drug-resistant (XDR) tuberculosis has increased in Kazakhstan over the past decade (2–8). The emergence of antimicrobial resistance in Kazakhstan took place in the 2000s after the collapse of the Soviet Union and was due to inadequate treatment strategies, interruptions in the drug supply, and the low socioeconomic status of the patients (9, 10).

Mycobacterium tuberculosis strain 3485_MTB was isolated from a sputum sample from a patient with clinically suspected tuberculosis in Nur-Sultan, Kazakhstan. The isolate was resistant to isoniazid, rifampin, streptomycin, ethambutol, capreomycin, kanamycin, and ofloxacin, which was confirmed through drug susceptibility testing using a Bactec MGIT 960 culture system (Becton, Dickinson), according to the manufacturer’s protocol. DNA was extracted using the traditional cetyltrimethylammonium bromide (CTAB) procedure (11). The quality of the DNA was checked using a Qubit double-stranded DNA (dsDNA) high-sensitivity (HS) assay kit (Thermo) and a Qubit 2.0 fluorometer (Thermo). One hundred nanograms of the total genomic DNA from M. tuberculosis strain 3485_MTB was sheared with a Bioruptor UCD-200 ultrasonicator (Diagenode). A barcoded library was prepared using an Ion Xpress Plus fragment library kit and an Ion Xpress barcode adapters 1-16 kit (Thermo), according to the manufacturer’s instructions. The sequencing was conducted on an Ion Torrent PGM sequencing platform using a Hi-Q sequencing kit (Thermo) and a 318 Chip (Thermo), as described previously (12). A total of 1,386,931 reads were produced, with a mean read length of 267 bp. The whole-genome shotgun sequencing data gave 79-fold coverage. Default parameters were used for all software, unless otherwise specified. The quality of the raw sequencing data was checked using FastQC v.0.11.9 (13). A total of 1,320 contigs, with an *N*_50_ of 28,454 bp, were *de novo* assembled using SPAdes v.3.1.0, with k-mer lengths of 21, 33, 55, 77, and 99 (14). The genome was annotated using the NCBI Prokaryotic Genome Annotation Pipeline v.4.10 (15). The draft whole-genome sequence of M. tuberculosis strain 3485_MTB has 4,836,003 bp, including 5,633 predicted coding sequences, 7 rRNAs, 52 tRNAs, and 4 noncoding RNA (ncRNA) genes, with a GC content of 64.07%. SAMtools v.1.10 (16) and GATK v.4.1.4 (17) were used to call single-nucleotide polymorphisms. The 3485_MTB genome harbors high-confidence mutations in the *rpoB* gene (Ser450Leu), *katG* gene (Ser315Thr), *rpsL* gene (Lys43Arg), *embB* gene (Met306Val), *gyrA* gene (Asp94Gly), and *eis* gene (G-14A promoter) associated with drug resistance (18). A comparison of the 3485_MTB genome data with the available genomic data for other XDR isolates will be helpful to reveal the genetic differences responsible for the pathogenicity and transmission patterns of 3485_MTB.

### Data availability.

This whole-genome shotgun sequencing project has been deposited in DDBJ/ENA/GenBank under accession no. WUJN00000000. The version described in this paper is the first version, WUJN01000000. The raw data from BioProject PRJNA596845 were submitted to the NCBI SRA under experiment accession no. SRR10742909.
